# Health emergencies and interoceptive sensibility modulate the perception of non-evidence-based drug use: Findings from the COVID-19 outbreak

**DOI:** 10.1371/journal.pone.0256806

**Published:** 2021-08-26

**Authors:** Gerardo Salvato, Daniela Ovadia, Alessandro Messina, Gabriella Bottini

**Affiliations:** 1 Department of Brain and Behavioral Sciences, University of Pavia, Pavia, Italy; 2 Cognitive Neuropsychology Centre, ASST “Grande Ospedale Metropolitano” Niguarda, Milano, Italy; 3 NeuroMi, Milan Center for Neuroscience, Milan, Italy; 4 Center for Ethics in Science and Journalism, Milan, Italy; KEMRI Wellcome Trust Research Programme, KENYA

## Abstract

Scientific evidence plays an important role in the therapeutic decision-making process. What happens when physicians are forced to make therapeutic decisions under uncertainty? The absence of scientific guidelines at the beginning of a pandemic due to an unknown virus, such as COVID-19, could influence the perceived legitimacy of the application of non-evidence-based therapeutic approaches. This paper reports on a test of this hypothesis, in which we administered an ad hoc questionnaire to a sample of 64 Italian physicians during the first wave of the COVID-19 pandemic in Italy (April 2020). The questionnaire statements regarding the legitimacy of off-label or experimental drugs were framed according to three different scenarios (Normality, Emergency and COVID-19). Furthermore, as the perception of internal bodily sensations (i.e., interoception) modulates the decision-making process, we tested participants’ interoceptive sensibility using the Multidimensional Assessment of Interoceptive Awareness (MAIA). The results showed that participants were more inclined to legitimate non-evidence-based therapeutic approaches in the COVID-19 and Emergency scenarios than the Normality scenario. We also found that scores on the MAIA Trusting subscale positively predicted this difference. Our findings demonstrate that uncertain medical scenarios, involving a dramatic increase in patient volume and acuity, can increase risk-taking in therapeutic decision-making. Furthermore, individual characteristics of health care providers, such as interoceptive ability, should be taken into account when constructing models to prevent the breakdown of healthcare systems in cases of severe emergency.

## 1. Introduction

Clinical decision-making is a complex cognitive process, based on the gathering of semeiotic and empirical evidence, which allows for the formulation of a diagnosis and the subsequent selection of specific treatments [1]. Each medical case may involve a critical amount of uncertainty regarding its outcome and, as such, implies a putative risk-taking component. Researchers, along with policymakers, have tried to improve and standardise the clinical decision-making process by implementing a set of rules captured under the umbrella term of “evidence-based medicine” (EBM) [2]. Since its introduction, a compendium of EBM guidelines has been produced to formalise the different medical procedures [3].

However, previous studies have shown that EBM represents only one of the components that guide medical decision-making, as this process is also influenced by other, more personal factors, such as the clinicians’ attitudes and opinions [4], cognitive biases [5] and the information environment. Interestingly enough, these individual influences profoundly affect medical practitioners, even in the presence of clear evidence-based recommendations. For instance, in common medical scenarios involving upper respiratory tract infections, prescribing rates and the appropriateness of prescriptions vary widely, which emphasises the role of a physician’s overconfidence in a prescription’s effects and their understanding of possible side effects [6]. Sorum and colleagues [7] have demonstrated that regret can affect clinical decision-making in uncertain situations, so that physicians may be prompted to over-assess patients, even in the absence of symptoms and when evidence-based guidelines discourage such practices. These authors observed that the likelihood of a physician ordering non-relevant medical exams for symptomless patients was associated with the regret they anticipated feeling if they had done nothing with an ill patient. This suggests that emotional components may intervene in clinical decisions together with medical knowledge. In particular, some studies have demonstrated the impact of stress and anxiety levels on decision-making in different categories of healthcare providers, such as surgeons [8, 9], dentists [10] and senior radiography students [11] (for a review, see [12]).

The emotional component affecting clinical decision-making could also comprise the perceived gravity of the patient’s clinical situation. In a recent study, Martìnez-Sanz and colleagues [13] investigated the clinical decision-making process during the COVID-19 pandemic. They demonstrated that heterogeneity of therapeutic decisions increased as the clinical scenario worsened, indicating that the clinical severity attenuated the physician’s tolerance for uncertainty and affected the decision-making process.

A growing body of evidence indicates that the decision-making process is also influenced by the ability to perceive one’s visceral signals [14–16], which is defined as “interoception” [17–19]. According to the Somatic Marker Hypothesis [20], interoceptive signals (e.g., elevated heart rate and sweating) may serve to guide decision-making efficiently, warning participants on possible outcomes. Indeed, automatically generated bodily responses (i.e., somatic signals) direct the subject’s attention towards “the negative outcome to which a given action may lead, and function as an automated alarm signal that says: Beware of danger ahead if you choose the option that leads to this outcome” ([21] p.123). Thus, individuals with accurate interoception would perform significantly better than those with lower interoception levels at tests exploring risk-taking under uncertainty [22]. Crucially, evidence regarding the relationship between decision-making and interoception among healthcare providers is scarce. Payne [23] used skin conductance response to obtain a physiological index in different samples during a clinical decision-making task. Specifically, the authors presented several computer-generated clinical scenarios to experienced nurses and nursing students. They found that the former group made better clinical decisions and also presented a higher skin conductance response.

Existing studies have mostly investigated clinical decision-making in the framework of common medical scenarios. There is limited evidence regarding medical decision-making during an emergency, when physicians are forced to act under uncertainty. Indeed, in such situations, reliable evidence-based approaches may not be available, which may increase the importance of individual orientation in decision-making. This was the case in the recent COVID-19 outbreak. The lack of resources and suitable medical treatments and the overwhelming and contradictory information regarding drug effectiveness during this health crisis has required healthcare providers to face critical situations and pushed them to make rapid and crucial decisions about patients’ treatment [24]. Clinicians have been forced to make decisions regarding resource allocation and treatments in an exceptional situation in the absence of evidence-based therapeutic approaches for COVID-19 patients. During the first few months of the pandemic, despite the lack of structured guidance regarding their efficacy, the use of many off-label drugs was proposed to treat the COVID-19 symptoms [25]. For instance, one of the best-known and most applied drugs in the clinical setting is hydroxychloroquine (HCQ), an antimalarial drug, which had previously been shown to be highly effective against avian influenza [26] and severe acute respiratory syndrome coronavirus (SARS-CoV) in vitro [27]. Despite these findings, many subsequent studies, such as the RECOVERY trial [28], demonstrated that in hospitalised patients with COVID-19, those who received HCQ did not have a lower incidence of death at 28 days compared to those who received the usual care. Despite the lack of an adequate evidence-based alternative, these treatments have been widely applied to COVID-19 patients. It seems that the pandemic, due to its exceptional nature, could have modulated the perceived legitimacy of the application of non-evidence-based therapeutic approaches. In this case, unexpected contextual factors such as the emergency and individual features such as interoception might play a more important role than external scientific guidelines (i.e., EBM) in therapeutic decision-making.

To test this hypothesis, we engaged a sample of 64 Italian physicians facing the pandemic at its beginning and assessed their perceptions of the legitimacy of prescribing various possible treatments in various scenarios through an ad hoc online questionnaire that was part of a more extensive survey. After completing a socio-demographic survey, participants indicated whether they agreed with the use of various off-label therapies for patients in three types of scenarios (Normality, Emergency and COVID-19). They also completed the Multidimensional Assessment of Interoceptive Awareness (MAIA) [29] as a measure of interoception. Finally, we collected a variety of information that could have influenced decision-making and interoception, such as anxiety level and perceived severity of illness.

We expected physicians to show a higher level of agreement with the use of off-label drugs during the COVID-19 scenario compared to the Emergency and Normal scenarios. Moreover, we hypothesised that the perceived legitimacy of off-label drugs would be modulated by physicians’ ability to detect visceral signals. Based on previous evidence that higher interoceptive sensibility predicts more conservative risk-taking behaviour when a human body is involved in the decision [16], we expected physicians with higher interoceptive competence to show reduced risk-taking in prescribing off-label drugs. Lastly, we expected that higher anxiety levels, boosted by a higher perceived severity of the pandemic, together with personal factors such as having infected acquaintances [30], would influence the relationship between interoception and the perceived legitimacy of off-label drugs used within the COVID-19 scenario.

## 2. Materials and methods

### 2.1 Participants

The online survey was advertised through the Italian Medical Council network from the 10^th^ to the 30^th^ of April 2020. Sixty-four Italian physicians (32 females and 32 males) completed the survey. Most of the sample (81.25%) had a PhD degree and/or medical specialisation, and around half of the sample (48.43%) came from the most affected regions in Italy by the COVID-19 pandemic. For a detailed description of the demographics of the sample, see Table 1. Following the Declaration of Helsinki, the experimental procedures were approved by the Ethical Committee of the Department of Brain and Behavioral Sciences, University of Pavia (n° 032). Potential participants were presented with all the study information before the survey began. At the end of the information page, they were required to indicate their willingness to participate in the study by clicking on the “agree” or “disagree” button. Once the potential participant had clicked on the button indicating that they had read the information and agreed to participate, they were directed to the research survey questionnaire.

**Table 1:** Demographics of the sample.

	Sample N (%)
**Sex**	
Males	32 (50%)
Females	32 (50%)
**Age**	
<35	10 (15.62%)
36–45	17 (26.56%)
46–55	10 (15.62%)
56–65	19 (29.68%)
66–75	8 (12.5%)
**Degree**	
Medical Specialty	12 (18.75%)
Doctorate	52 (81.25%)

### 2.2 Measures

#### 2.2.1 Socio-demographic and COVID-19 questionnaires

At the beginning of the survey, respondents completed a socio-demographic questionnaire. Information on gender, age and marital status were collected, along with the degree of education and medical speciality. Because there is evidence that personal factors related to the pandemic affect stress levels [30], we reasoned that such factors could also affect the perceived legitimacy of off-label therapeutic approaches. Thus, we collected additional measures on this topic. Responders were required to state how many patients affected by COVID-19 they had treated. Furthermore, they were required to indicate their perception of the severity of the disease and their level of concern about contracting the disease. Both responses were expressed on a 6-point Likert scale ranging from 0 = “Not at all” to 5 = “Extremely”.

#### 2.2.2 Legitimacy questionnaire

To investigate respondents’ perceptions of the legitimacy of non-evidence-based therapeutic approaches, we developed a new questionnaire. Five different statements were included, each framed in three different scenarios (Normality, Emergency and COVID-19) for a total number of 15 items. For each triplet, the main clause of the sentence remained constant in the three conditions, while the subordinate clause, at the beginning or end of the sentence, specified the scenario in which it was framed (see S1 File for the complete list of sentences). All the statements related to the prescription and/or recommendation by institutions of non-evidence-based treatments. The extent to which the participant agreed with each of the statements was expressed on a Likert scale ranging from 0 = “Totally disagree” to 5 = “Totally agree”. A total of 15 statements were presented in a randomised order across participants.

#### 2.2.3 Interoception

Participants’ interoceptive sensibility (the self-report dimension of interoception) was assessed through the Italian version of the MAIA [29, 31]. The questionnaire consists of 32 items covering the subjective perception of internal bodily signals for different areas. Responses are given on a 6-point Likert scale ranging from 0 = “Never” to 5 = “Always”. For the scoring, eight measures are calculated from as many subscales that correspond to distinct dimensions of interoceptive sensibility, which makes it possible to differentiate between interoception features. Higher scores on each subscale indicate a better ability to perceive bodily signals and changes therein.

#### 2.2.4 Anxiety

As anxiety levels have been shown to modulate interoception and decision-making [32], we also quantified this component through the State-Trait Anxiety Inventory X (STAI X) [33]. The inventory comprises two scales of 20 items, each assessing state anxiety (i.e., anxiety level perceived during the test itself) and trait anxiety (i.e., anxiety level perceived generally). Responses to both scales are given on a 5-point Likert scale ranging from 0 = “Not at all” to 4 = “Very much so” for the first scale and from 0 = “Almost never” to 4 = “Almost always” for the second one. The resulting scores for the state and trait anxiety were computed by averaging subjects’ responses to the pertinent items as described in the manual.

All questionnaires were hosted on SurveyMonkey (Inc. San Mateo, California, USA www.surveymonkey.com), and the different tests were administered in a randomised order across participants.

### 2.3 Statistical analysis plan

Statistical analyses were conducted using SPSS 20 (Statistical Package for Social Science, Chicago, Illinois) and JAMOVI [34]. Firstly, a reliability analysis was carried out on the items included in the Legitimacy questionnaire.

Then, to study medical decision-making patterns in relation to the experimental conditions, we used a generalised linear mixed model. Condition (Normality, Emergency, COVID-19) was modelled as a fixed factor. The agreement scores were modelled as the target variable, for which a multinomial distribution with a cumulative logit function was adopted. A random intercept modelled on the subjects was included.

Thirdly, we investigated the extent to which interoception modulates the perceived legitimacy of non-evidence-based treatments in the case of a COVID-19 scenario compared to a Normal scenario. To this aim, for each subject, we calculated a score for each of the Legitimacy questionnaire condition by averaging responses at items 1,4,7,10,13 for the Normality scenario, items 2,5,8,11,14 for the Emergency scenario and items 3,6,9,12,15 for the COVID-19 scenario. Then, we generated a *legitimacy index*, calculated as the difference between the COVID-19 and Normality scenario scores. Higher *legitimacy index* values indicate higher perceived legitimacy in the case of the COVID-19 compared to the Normality scenario. To investigate the impact of interoception on this index, we ran a linear regression analysis with the eight MAIA subscales as predictors and *legitimacy index* scores as the dependent variable. Participants’ age and gender were included as covariates.

Lastly, we explored the potential effects of other psychological factors on the *legitimacy index*. To this aim, we ran a Pearson’s partial correlation analysis between the *legitimacy index* and the (i) number of COVID-19 patients treated, (ii) perceived severity and (iii) preoccupation with the COVID-19 outbreak, (iv) anxiety level, controlling for the participants’ age and gender.

## 3. Results

Socio-demographic variables and responses to the COVID-19 questionnaire are shown in Tables 1 and 2. Out of the 64 respondents, 50% were women. Most of the respondents had already treated at least one COVID-19 patient (76.56%). Results from the STAI-X are reported in Table 3.

**Table 2:** Number of responses for each alternative in the COVID-19 questionnaire.

	Sample N (%)
**"How many COVID-19 patients did you treat?"**	
None	15 (23,43%)
1–5	14 (21,87%)
6–15	11 (17,18%)
>15	24 (37,5%)
**"How serious is COVID-19 for health?"**	
Not at all serious	1 (1,56%)
Slightly serious	2 (3,12%)
Moderately serious	14 (21,87%)
Very serious	27 (42,18%)
Extremely serious	20 (31,25%)
**"How much are you worried to become infected with COVID-19?"**	
Not at all worried	3 (4,68%)
Slightly worried	18 (28,12%)
Moderately worried	33 (51,56%)
Very worried	9 (14,06%)
Extremely worried	1 (1,56%)

**Table 3:** Mean score and standard deviation for the State-Trait Anxiety Inventory.

	Mean score	St. Dev.
**STAI—State Anxiety**	42.84	10.54
**STAI—Trait Anxiety**	39	8.53

The analysis of the legitimacy questionnaire items showed good internal consistency (Cronbach’s α = 0.835). Most items appeared to be worthy of retention and resulted in a decrease in the alpha value if deleted.

We also found that the scenario types modulated the perceived legitimacy of non-evidence-based therapeutic approaches. The generalised linear mixed model results showed a main effect of Condition (*F*_*(*2,6)_ = 10.974, *p* = 0.010), indicating that participants were more inclined to legitimate non-evidence-based therapeutic approaches in the COVID-19 scenario compared to the Normality scenario (*t* = -3.915, *p* = 0.008). There was no difference between the Emergency and COVID-19 scenarios (*t* = 0.422, *p* = 0.688).

**Fig 1:**
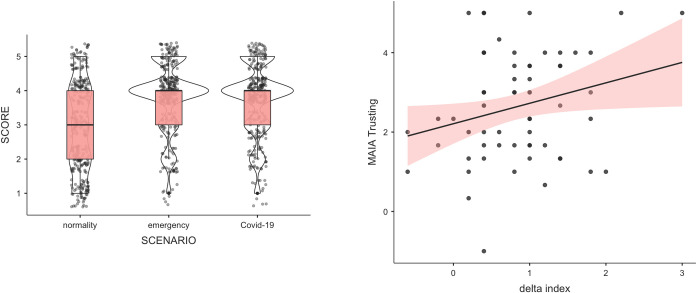
Results. The left panel shows the distribution of raw scores in the three conditions. The right panel shows the results of the regression analysis between the subscale of the MAIA (Trusting) and the delta values of the *legitimacy index* (difference between perceived legitimacy scores in the COVID-19 and Normality scenarios).

Concerning the regression analysis investigating the relationship between the *legitimacy index* and interoception, all the assumptions were met. The residuals were normally distributed (*Shapiro–Wilk* = 0.975; *p* = 0.225); the residuals were independent (*Durbin–Watson* = 1.89), and for all the predictors, the VIF value was < 10, indicating the absence of multicollinearity. We found that the more physicians experienced their own bodies as safe and trustworthy, the higher were their perceptions of the legitimacy of non-evidence-based therapeutic approaches. The results showed that the overall model explained a significant amount of the variance in the legitimacy index results (*R^2^* = 0.142, *F*_(10, 53)_ = 2.04, *p* = 0.047); in particular, the MAIA Trusting subscale positively predicted the *legitimacy index* (*b* = 0.362, *t* = 2.198, *p* = 0.032) (Fig 1).

Lastly, the *legitimacy index* did not correlate with number of COVID-19 patients treated (*r*_(62)_ = 0.240, *p* = 0.60), perceived severity of (*r*_(62)_ = 0.103, *p* = 0.425) and concern about the COVID-19 outbreak (*r*_(62)_ = -0.068, *p* = 0.597) or the participants’ state (*r*_(62)_ = -0.057, *p* = 0.658) and trait (*r*_(62)_ = 0.045, *p* = 0.730) anxiety levels.

## 4. Discussion

Healthcare providers continuously make clinical decisions, either for diagnosis or for treatment. Scientific evidence is an important contributor to this process, as it reduces the uncertainty about the possible outcomes [35]. However, reliable scientific evidence is not always available or adequately communicated, as occurred during the COVID-19 outbreak in early 2020 in Italy, which has also posed many challenges to national health services worldwide. This exceptional situation forced medical institutions and physicians to make critical choices about the allocation of already limited resources [36]. Moreover, the lack of an effective medical treatment of the virus favoured off-label, non-evidence-based treatments [37]. In this study, we investigated whether different medical scenarios modulated the perceived legitimacy of non-evidence-based therapeutic approaches. Moreover, we tested the influence of biological and psychological factors on such behaviour.

Our findings demonstrated that the COVID-19 pandemic and, in general, medical emergency scenarios pushed physicians to consider non-evidence-based treatments as legitimate possibilities. We speculated that physicians’ responses were influenced by different semantic descriptions of the same issue in alignment with the so-called “framing effect” [38], according to which people tend to make decisions based on their perception of the potential losses and gains rather than on the outcome itself. Interpreting our results, due to the high mortality rate caused by the pandemic, it might be hypothesised that physicians based their decisions on the potential losses rather than the potential gains. In other words, they viewed the risk of prescribing non-EBM treatments more favourably than the risk of not saving the patient’s life. In the same vein, in the clinical setting, previous studies [6] have demonstrated that the rate of prescription depends on how a decision is framed. More specifically, emergency department clinicians who considered antibiotics essentially harmless showed higher prescription rates of antibiotics to treat pneumonia compared to clinicians who considered antibiotics potentially harmful [6]. Considering the COVID-19 pandemic, in a recent study [13], physicians were asked which treatment they would have applied to manage five increasingly severe medical cases presenting with symptoms congruent with COVID-19 infection. The results showed that the higher the clinical severity, the higher the number of chosen treatments combinations, although evidence about the effectiveness of such therapeutic strategies has been scarce. Indeed, in the worst clinical cases, this resulted in the prescription of up to six different drugs, among which the authors identified HCQ and other off-label medications.

Our results also demonstrated that the physicians relied on internal signals to support decision-making. Contrary to our initial hypothesis, we found that the higher the physician’s trust in their bodily signals (as indicated by their score on the Trusting subscale of the MAIA), the greater their propensity to consider off-label drugs a worthy therapeutic choice. The Trusting subscale reflects the subject’s tendency to consider the perception of bodily sensations as helpful for decision-making and health [29]. Consistently, it has been correlated with measures related to body-listening and self-awareness abilities that are useful in guiding decision-making, such as the Listening subscale in the Body Responsiveness Questionnaire [29, 39]. Furthermore, it has been associated with emotion regulation and awareness [40] and low anxiety levels [29]. These results lead to the speculation that when clinical decision-making is mandatory but essential knowledge is lacking, physicians may rely on internal bodily signals and information to assess the situation and guide their behaviour. Our results also suggest that anxiety level or perceived severity of illness did not modulate the relationship between interoception and the perceived legitimacy of off-label drugs. This finding may be specific to the category of the population that was studied, that is, physicians, who might have better strategies than non-physicians to cope with their own anxiety levels. Indeed, coping strategies may play an important role in mediating the outcomes of stressful events, as demonstrated by studies of the severe acute respiratory syndrome outbreak of 2003 [41].

The association between bodily signals and clinical decision-making has already been pointed out in healthcare providers. Payne [23] demonstrated that more experienced nurses displayed a higher skin conductance response than nursing students when deciding on a course of treatment for clinical cases. Accordingly, the author concluded that physiological processes might have informed clinical decision-making processes in this specific task. Similar results have also been found in a study focused on a different professional category [42], namely market traders, to corroborate the relationship between implicit body components and complex cognitive processes. This study showed that traders had better interoceptive performance than non-traders on the heartbeat detection task. Furthermore, traders’ interoceptive ability predicted their relative profitability and, strikingly, how long they survived in the financial markets.

To conclude, clinical uncertainty due to unexpected and dangerous situations such as the COVID-19 pandemic may increase the prevalence of divergent medical decisions. This appears to be related to a conflict between the reasonable tendency to adapt to standard (i.e., EBM) guidelines and the individual biological features of professional figures (physicians), such as interoceptive competence. Our results, indeed, demonstrate that physicians’ reliance on internal signals supported the decision-making. As these individual characteristics may sensibly guide physicians and other healthcare providers’ decision-making, they should be taken into account when constructing models to prevent the occurrence of medical errors and the breakdown of healthcare systems in cases of severe emergency.

### 4.1 Limitations

This study presents some limitations. Since the World Health Organization’s guidelines for preventing COVID-19 transmission strongly discourage contact and proximity between people, especially in indoor settings, we decided to rely on self-report measures to explore both the perceived legitimacy of off-label or experimental drugs and interoception. It would be relevant to complement this study by exploring which pharmacological treatments and possible combinations were prescribed to the COVID-19 patients treated by the sample of physicians enrolled in the current study. Concerning the interoception, also in this case, a more objective measure would detail its role in the decision-making process better. Although the MAIA questionnaire is widely used to measure interoception [31, 40, 43, 44], future studies should investigate the role of more objective measures of interoception (i.e., interoceptive accuracy) in the clinical decision-making process during emergencies.

## Supporting information

S1 File Legitimacy questionnaire. (DOCX)

## References

[1] Tiffen J, Corbridge SJ, Slimmer L. Enhancing Clinical Decision Making: Development of a Contiguous Definition and Conceptual Framework. Journal of Professional Nursing. 2014. doi: 10.1016/j.profnurs.2014.01.006 25223288

[2] Djulbegovic B, Guyatt GH. Progress in evidence-based medicine: a quarter century on. The Lancet. 2017. doi: 10.1016/S0140-6736(16)31592-6 28215660

[3] Vere J, Gibson B. Evidence-based medicine as science. Journal of Evaluation in Clinical Practice. 2019. doi: 10.1111/jep.13090 30575209

[4] Williams N, Kogan R. Factors associated with evidence-based decision-making among patients and providers. Journal of Comparative Effectiveness Research. 2019. doi: 10.2217/cer-2018-0152 31290682

[5] Pines JM, Strong A. Cognitive Biases in Emergency Physicians: A Pilot Study. Journal of Emergency Medicine. 2019. doi: 10.1016/j.visj.2019.100571 31126674

[6] Klein EY, Martinez EM, May L, Saheed M, Reyna V, Broniatowski DA. Categorical Risk Perception Drives Variability in Antibiotic Prescribing in the Emergency Department: A Mixed Methods Observational Study. Journal of General Internal Medicine. 2017. doi: 10.1007/s11606-017-4099-6 28634909 PMC5602760

[7] Sorum PC, Shim J, Chasseigne G, Bonnin-Scaon S, Cogneau J, Mullet E. Why do primary care physicians in the United States and France order prostate-specific antigen tests for asymptomatic patients? Medical Decision Making. 2003. doi: 10.1177/0272989X03256010 12926580

[8] Dale W, Hemmerich J, Ghini EA, Schwarze ML. Can Induced Anxiety from a Negative Earlier Experience Influence Vascular Surgeons’ Statistical Decision-Making? A Randomized Field Experiment with an Abdominal Aortic Aneurysm Analog. Journal of the American College of Surgeons. 2006;203: 642–652. doi: 10.1016/j.jamcollsurg.2006.07.020 17084325

[9] Wetzel CM, Kneebone RL, Woloshynowych M, Nestel D, Moorthy K, Kidd J, et al. The effects of stress on surgical performance. The American Journal of Surgery. 2006;191: 5–10. doi: 10.1016/j.amjsurg.2005.08.034 16399098

[10] Chipchase SY, Chapman HR, Bretherton R. A study to explore if dentists’ anxiety affects their clinical decision-making. Br Dent J. 2017;222: 277–290. doi: 10.1038/sj.bdj.2017.173 28232686

[11] Cumming SR, Harris LM. The impact of anxiety on the accuracy of diagnostic decision-making. Stress and Health. 2001;17: 281–286. doi: 10.1002/smi.909

[12] Kozlowski D, Hutchinson M, Hurley J, Rowley J, Sutherland J. The role of emotion in clinical decision making: an integrative literature review. BMC Med Educ. 2017;17: 255. doi: 10.1186/s12909-017-1089-7 29246213 PMC5732402

[13] Martínez-Sanz J, Pérez-Molina JA, Moreno S, Zamora J, Serrano-Villar S. Understanding clinical decision-making during the COVID-19 pandemic: A cross-sectional worldwide survey. EClinicalMedicine. 2020. doi: 10.1016/j.eclinm.2020.100539 32923995 PMC7480231

[14] Toplak ME, Sorge GB, Benoit A, West RF, Stanovich KE. Decision-making and cognitive abilities: A review of associations between Iowa Gambling Task performance, executive functions, and intelligence. Clinical Psychology Review. 2010. doi: 10.1016/j.cpr.2010.04.002 20457481

[15] Fischhoff B, Broomell SB. Judgment and decision making. Annual Review of Psychology. 2020. doi: 10.1146/annurev-psych-010419-050747 31337275

[16] Salvato G, De Maio G, Bottini G. Interoceptive sensibility tunes risk-taking behaviour when body-related stimuli come into play. Scientific Reports. 2019;9: 2396. doi: 10.1038/s41598-019-39061-0 30787367 PMC6382876

[17] Craig a. D. How do you feel? Interoception: the sense of the physiological condition of the body. Nature Reviews Neuroscience. 2002;3: 655–666. doi: 10.1038/nrn894 12154366

[18] Critchley HD, Garfinkel SN. Interoception and emotion. Current Opinion in Psychology. 2017;17: 7–14. doi: 10.1016/j.copsyc.2017.04.020 28950976

[19] Salvato G, Richter F, Sedeño L, Bottini G, Paulesu E. Building the bodily self‐awareness: Evidence for the convergence between interoceptive and exteroceptive information in a multilevel kernel density analysis study. Human Brain Mapping. 2020;41: 401–418. doi: 10.1002/hbm.24810 31609042 PMC7268061

[20] Bechara A, Damasio H, Damasio AR. Emotion, decision making and the orbitofrontal cortex. Cerebral Cortex. 2000. doi: 10.1093/cercor/10.3.295 10731224

[21] Damasio AR. Descartes’ error: Emotion, rationality and the human brain. New York: Putnam. 1994; 352–352.

[22] Werner NS, Jung K, Duschek S, Schandry R. Enhanced cardiac perception is associated with benefits in decision-making. Psychophysiology. 2009;46: 1123–1129. doi: 10.1111/j.1469-8986.2009.00855.x 19558399

[23] Payne LK. Physiological differences during decision making between experienced nurses and nursing students: A pilot study. Journal of Nursing Education. 2013. doi: 10.3928/01484834-20131017-02 24127613

[24] Mannelli C, Mannelli C. Whose life to save? Scarce resources allocation in the COVID-19 outbreak. Journal of Medical Ethics. 2020. doi: 10.1136/medethics-2020-106227 32277018

[25] Khan S, Gionfriddo MR, Cortes-Penfield N, Thunga G, Rashid M. The trade-off dilemma in pharmacotherapy of COVID-19: systematic review, meta-analysis, and implications. Expert Opinion on Pharmacotherapy. 2020. doi: 10.1080/14656566.2020.1792884 32752970

[26] Yan Y, Zou Z, Sun Y, Li X, Xu KF, Wei Y, et al. Anti-malaria drug chloroquine is highly effective in treating avian influenza A H5N1 virus infection in an animal model. Cell Research. 2013. doi: 10.1038/cr.2012.165 23208422 PMC3567830

[27] Vincent MJ, Bergeron E, Benjannet S, Erickson BR, Rollin PE, Ksiazek TG, et al. Chloroquine is a potent inhibitor of SARS coronavirus infection and spread. Virology Journal. 2005. doi: 10.1186/1743-422X-2-69 16115318 PMC1232869

[28] Group RC. Effect of hydroxychloroquine in hospitalized patients with Covid-19. New England Journal of Medicine. 2020;383: 2030–2040. 10.1056/NEJMoa2022926 PMC7556338 33031652

[29] Mehling WE, Price C, Daubenmier JJ, Acree M, Bartmess E, Stewart A. The Multidimensional Assessment of Interoceptive Awareness (MAIA). PLoS ONE. 2012. doi: 10.1371/journal.pone.0048230 23133619 PMC3486814

[30] Mazza C, Ricci E, Biondi S, Colasanti M, Ferracuti S, Napoli C, et al. A Nationwide Survey of Psychological Distress among Italian People during the COVID-19 Pandemic: Immediate Psychological Responses and Associated Factors. International Journal of Environmental Research and Public Health. 2020. doi: 10.3390/ijerph17093165 32370116 PMC7246819

[31] Calì G, Ambrosini E, Picconi L, Mehling WE, Committeri G. Investigating the relationship between interoceptive accuracy, interoceptive awareness, and emotional susceptibility. Frontiers in Psychology. 2015;6: 1–13. doi: 10.3389/fpsyg.2015.00001 26379571 PMC4547010

[32] Solano López AL, Moore S. Dimensions of Body-Awareness and Depressed Mood and Anxiety. Western Journal of Nursing Research. 2019. doi: 10.1177/0193945918798374 30178716

[33] Spielberger CD, Gorsuch RL, Lushene RE. STAI manual for the state-trait anxiety inventory. Self-Evaluation Questionnaire. MANUAL. 1970.

[34] Jamovi. The JAMOVI project. [Computer software]. 2020.

[35] Molony DA, Samuels JA. Evidence-Based Medicine: A Strategy to Reduce Clinical Uncertainty, Resulting in Improved Patient Outcomes and Population Health and Reduced Cost Through Improvements in Care. Advances in Chronic Kidney Disease. 2012. doi: 10.1053/j.ackd.2012.01.005 22364794

[36] Cesari M, Proietti M. COVID-19 in Italy: Ageism and Decision Making in a Pandemic. Journal of the American Medical Directors Association. 2020. doi: 10.1016/j.jamda.2020.03.025 32334771 PMC7118618

[37] Krouse HJ. Whatever Happened to Evidence-Based Practice During COVID-19? Otolaryngology—Head and Neck Surgery (United States). 2020. doi: 10.1177/0194599820930239 32423297

[38] Tversky A, Kahneman D. The framing of decisions and the psychology of choice. Science. 1981. doi: 10.1126/science.7455683 7455683

[39] Daubenmier JJ. The relationship of yoga, body awareness, and body responsiveness to self-objectification and disordered eating. Psychology of Women Quarterly. 2005. doi: 10.1111/j.1471-6402.2005.00236.x 26451071 PMC4594833

[40] Brown TA, Berner LA, Jones MD, Reilly EE, Cusack A, Anderson LK, et al. Psychometric Evaluation and Norms for the Multidimensional Assessment of Interoceptive Awareness (MAIA) in a Clinical Eating Disorders Sample. European Eating Disorders Review. 2017. doi: 10.1002/erv.2532 28714581

[41] Phua DH, Tang HK, Tham KY. Coping responses of emergency physicians and nurses to the 2003 severe acute respiratory syndrome outbreak. Academic emergency medicine. 2005;12: 322–328. doi: 10.1197/j.aem.2004.11.015 15805323

[42] Kandasamy N, Garfinkel SN, Page L, Hardy B, Critchley HD, Gurnell M, et al. Interoceptive Ability Predicts Survival on a London Trading Floor. Scientific Reports. 2016. doi: 10.1038/srep32986 27641692 PMC5027524

[43] Salvato G, Romano D, De Maio G, Bottini G. Implicit mechanisms of body image alterations: The covert attention exposure effect. Attention, Perception, and Psychophysics. 2020;82. doi: 10.3758/s13414-019-01921-2 31808112

[44] Ferentzi E, Olaru G, Geiger M, Vig L, Köteles F, Wilhelm O. Examining the factor structure and validity of the multidimensional assessment of interoceptive awareness. Journal of Personality Assessment. 2020; 1–10. doi: 10.1080/00223891.2020.1813147 32955947

