# Supplementary material for: Health emergencies and interoceptive sensibility modulate the perception of non-evidence-based drug use: Findings from the COVID-19 outbreak
Source: PLoS One. 2021 Aug 26;16(8):e0256806. doi: 10.1371/journal.pone.0256806 (PMC8389377; doi:10.1371/journal.pone.0256806)
Supplement: S1 File — (DOCX) [file pone.0256806.s001.docx]

*Health emergencies and interoceptive sensibility modulate the perception of non-evidence-based drug use: findings from the COVID-19 outbreak.*

Gerardo Salvato^1,2,3^; Daniela Ovadia^1,3,4^; Alessandro Messina^1^; Gabriella Bottini^1,2,3^

^1^ Department of Brain and Behavioral Sciences, University of Pavia, Pavia, Italy.

^2^ Cognitive Neuropsychology Centre, ASST “Grande Ospedale Metropolitano” Niguarda, Milano, Italy.

^3^ NeuroMi, Milan Center for Neuroscience, Milan, Italy.

^4^ Center for Ethics in Science and Journalism, Milan

**Legitimacy Questionnaire:**

1) In ordinary clinical practice, physicians should be allowed to prescribe any drugs he or she deems useful to the best of his or her knowledge and beliefs.

2) In the event of a medical emergency, physicians should be allowed to prescribe any drugs deemed useful to the best of our knowledge and beliefs.

3) In the event of COVID-19, physicians should be allowed to prescribe any drugs deemed useful to the best of our knowledge and beliefs.

4) In ordinary clinical practice, it is legitimate to prescribe “intelligent” drugs, such as monoclonal antibodies, antiretroviral drugs or drugs directed against molecular targets, in the presence of the molecular target, even in the absence of an efficacy study.

5) In the event of a medical emergency, it is legitimate to prescribe “intelligent” drugs, such as monoclonal antibodies, antiretroviral drugs or drugs directed against molecular targets, in the presence of the molecular target, even in the absence of an efficacy study.

6) In the event of COVID-19, it is legitimate to prescribe “intelligent” drugs, such as monoclonal antibodies, antiretroviral drugs or drugs directed against molecular targets, in the presence of the molecular target, even in the absence of an efficacy study.

7) In ordinary clinical practice, the prescription of drugs with known toxicity and side effects, as in the case of hydroxychloroquine, is legitimate.

8) In the event of a medical emergency, the prescription of drugs with known toxicity and side effects, as in the case of hydroxychloroquine, is legitimate.

9) In the event of COVID-19, the prescription of drugs with known toxicity and side effects, as in the case of hydroxychloroquine, is legitimate.

10) In ordinary clinical practice, I believe it is correct that institutions (Ministry of Health, regions, scientific societies) may recommend the use of an off-label or investigational medicine.

11) In the event of a medical emergency, I believe it is correct that institutions (Ministry of Health, regions, scientific societies) may recommend the use of an off-label or investigational medicine.

12) In the event of COVID-19, I believe it is correct that institutions (Ministry of Health, regions, scientific societies) may recommend the use of an off-label or investigational medicine.

13) In ordinary clinical practice, I believe it is correct that institutions (Ministry of Health, regions, scientific societies) may recommend lifestyles, behaviours, or the use of personal protective equipment even in the absence of clear evidence-based efficacy.

14) In the event of a medical emergency, I believe it is correct that institutions (Ministry of Health, regions, scientific societies) may recommend lifestyles, behaviours, or the use of personal protective equipment even in the absence of clear evidence-based efficacy.

15) In the event of COVID-19, I believe it is correct that institutions (Ministry of Health, regions, scientific societies) may recommend lifestyles, behaviours, or the use of personal protective equipment even in the absence of clear evidence-based efficacy.
